# A systematic review of Vaccine Breakthrough Infections by SARS-CoV-2 Delta Variant

**DOI:** 10.7150/ijbs.68973

**Published:** 2022-01-01

**Authors:** Mengxin Zhang, Ying Liang, Dongsheng Yu, Bang Du, Weyland Cheng, Lifeng Li, Zhidan Yu, Shuying Luo, Yaodong Zhang, Huanmin Wang, Xianwei Zhang, Wancun Zhang

**Affiliations:** 1 Henan Key Laboratory of Children's Genetics and Metabolic Diseases, Children's Hospital Affiliated to Zhengzhou University, Henan Children's Hospital, Zhengzhou Children's Hospital, Zhengzhou 450018, China; 2 Department of Chinese Medicine, The First Affiliated Hospital of Zhengzhou University, Zhengzhou, 450000, China

**Keywords:** SARS-CoV-2, COVID-19, Delta variant, Vaccine, Breakthrough infection, S protein

## Abstract

Vaccines are proving to be highly effective in controlling hospitalization and deaths associated with severe acute respiratory syndrome coronavirus 2 (SARS-CoV-2) infection, as shown by clinical trials and real-world evidence. However, a deadly second wave of coronavirus disease 2019 (COVID-19), infected by SARS-CoV-2 variants, especially the Delta (B.1.617.2) variant, with an increased number of post-vaccination breakthrough infections were reported in the world recently. Actually, Delta variant not only resulted in a severe surge of vaccine breakthrough infections which was accompanied with high viral load and transmissibility, but also challenged the development of effective vaccines. Therefore, the biological characteristics and epidemiological profile of Delta variant, the current status of Delta variant vaccine breakthrough infections and the mechanism of vaccine breakthrough infections were discussed in this article. In addition, the significant role of the Delta variant spike (S) protein in the mechanism of immune escape of SARS-CoV-2 was highlighted in this article. In particular, we further discussed key points on the future SARS-CoV-2 vaccine research and development, hoping to make a contribution to the early, accurate and rapid control of the COVID-19 epidemic.

## Introduction

The coronavirus disease 2019 (COVID-19) caused by severe acute respiratory syndrome coronavirus 2 (SARS-CoV-2) infections has resulted in more than 245 million infected cases and nearly 4.97 million deaths worldwide as of October 27, 2021, causing serious negative impact on global health and economic development 1-3. Vaccines have been developed and rapidly implemented in order to control the global spread of COVID-19, among which consist of seven vaccines authorized by the World Health Organization (WHO) listed under emergency use listing (EUL) 4, 5. Clinical trials have shown that the efficacy of different vaccines can range from 50% to 95% 6-8. Undoubtedly, COVID-19 vaccines have played an important role in controlling the spread of COVID-19, especially in reducing the incidence of severe COVID-19 cases or deaths caused by SARS-CoV-2 in practical application 9, 10. Nevertheless, the number of post-vaccinal breakthrough infections, especially caused by the Delta (B.1.617.2) variant, is increasing exponentially worldwide. Rising numbers of breakthrough infections not only have a significant negative impact on global public health security, but also increases speculation on the possibility of vaccine failure, causing panic in society 11, 12. Accordingly, we examined the biological characteristics and epidemiological profile of Delta variant, the current status of Delta variant vaccine breakthrough infection and the mechanism of vaccine breakthrough infection in this article.

**Figure 1:**
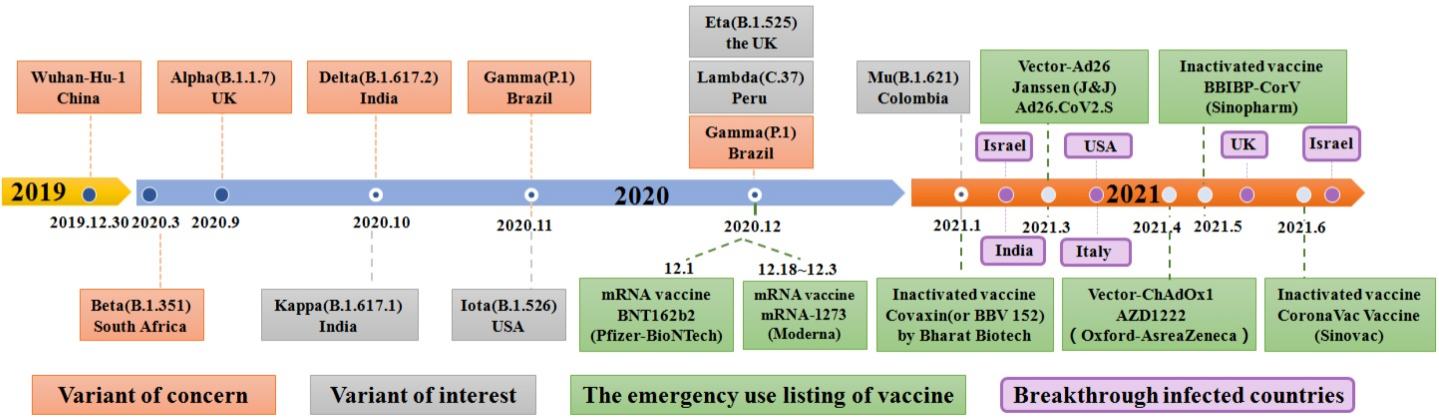
A timeline depicting the origin of SARS-CoV-2 VOC (Variant of Concern) and SARS-CoV-2 VOI (Variant of Interest), as well as when SARS-CoV-2 vaccines are authorized to be added to the EUL (emergency use listing) of WHO and the SARS-CoV-2 vaccine breakthrough infection countries.

**Figure 2:**
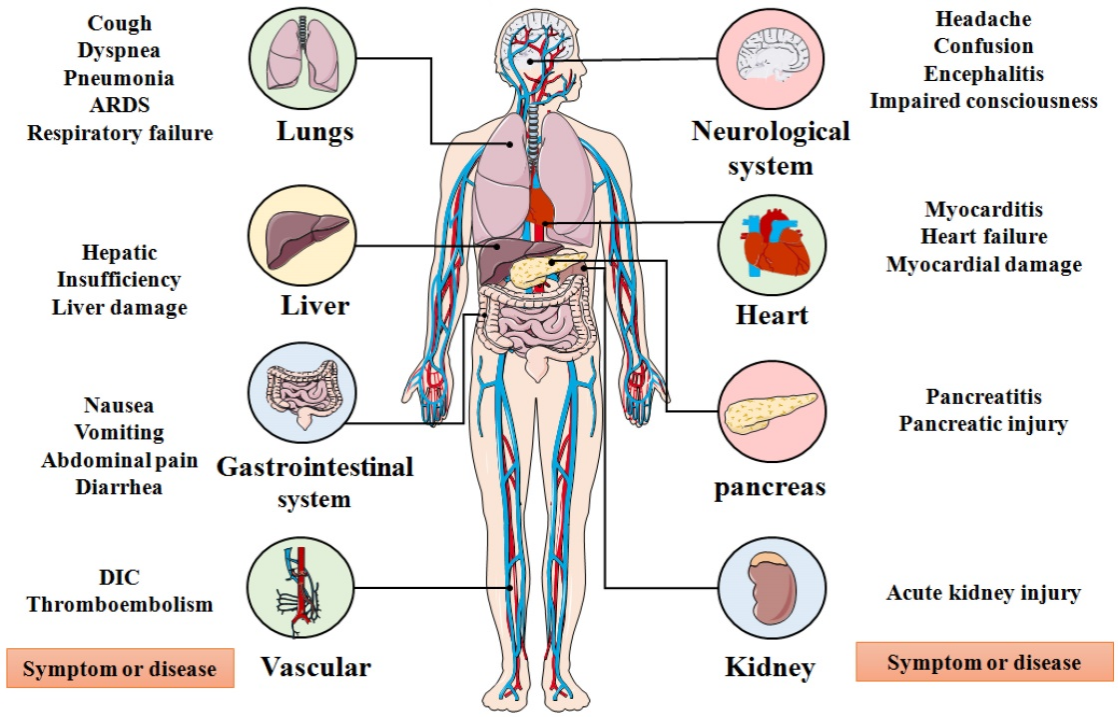
Organs affected by SARS-CoV-2 and corresponding systemic symptoms and diseases. ARDS: Acute respiratory distress syndrome; DIC: Disseminated intravascular coagulation.

SARS-CoV-2, an enveloped, positive stranded ribonucleic acid (RNA) virus, was first reported in December 2019 and after which infections rose exponentially around the world 6, 13, 14. Recently, SARS-CoV-2 rapidly evolved into several deadly variants of concern (VOC) from September to December 2020, including the Alpha (B.1.1.7) variant, Beta (B.1.35) variant, Gamma (B.1.1.28.1) variant, and Delta (B.1.617.2) variant (**Figure 1**) 15-20. Since October 2020, the number of variants of interests (VOI) has continuously evolved which includes the Kappa (B.1.617.1) variant, the Iota (B.1.526) variant, the Eta (B.1.525) variant, the Lambda (C.37) variant, and the Gamma (P.1) variant 21. Among existing VOCs and VOIs, the Delta variant, first detected in October 2020, was reported in the Maharashtra state of India, surpassing other variants of the same lineage where it became the most widespread and rapidly-spreading variant among nearly 200 other countries 21-27. Clinical research showed that the emergence of the Delta variant can incite damage in the lung, liver, gastrointestinal system, vascular, neurological system, heart, pancreas, kidneys and other organs of infected people, leading to a series of clinical manifestations, such as cough, pneumonia, dyspnea, acute respiratory distress syndrome (ARDS), respiratory failure, liver damage, abdominal pain, disseminated intravascular coagulation (DIC), myocardial damage, pancreatic injury and acute kidney injury as other SARS-CoV-2 variants **(Figure 2)** 28, 29. In particular, the Delta variant also displays a series of unique physiological characteristics and clinical features compared to other SARS-CoV-2 variants, such as high viral load, strong transmissibility and resistance against existing monoclonal antibodies therapy 30-34. These features of the Delta Variant have led to higher transmission characteristics and mortality compared with other SARS-COV-2 variants.

## Status of COVID-19 vaccine development

**Figure 3:**
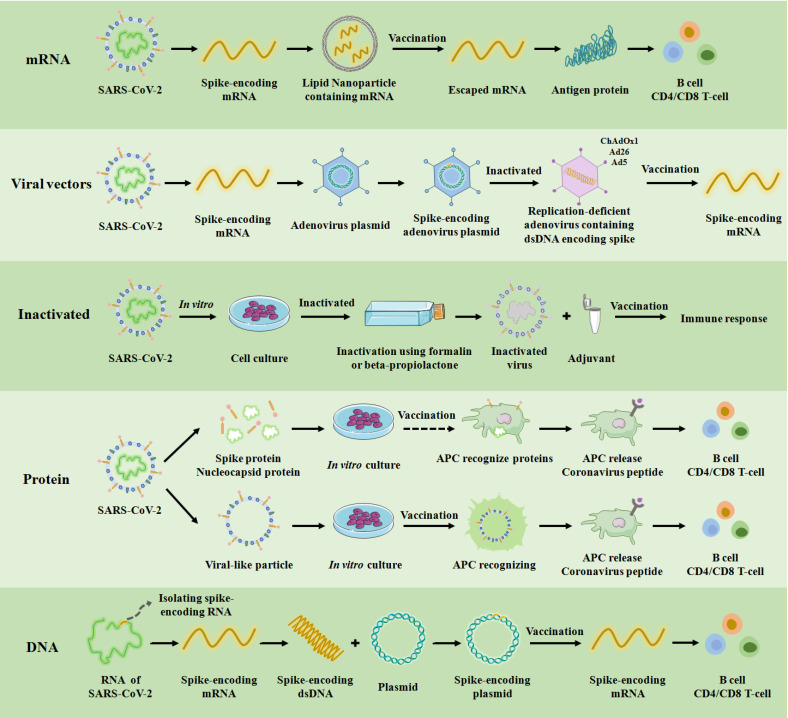
COVID-19 vaccine platforms. A schematic of the targets, production process and immune mechanism of the underlying five types of COVID-19 vaccines currently available in the clinic. APC: antigen presenting cell. ChAdOx1, Ad26, and Ad5 are among the 100 serotypes of adenovirus, and these three have been used as non-replicating adenovirus vectors in clinical practice for vaccination.

Since the first reports of infections caused by the novel coronavirus, there has been an unprecedented global effort to design, manufacture and test multiple vaccines against SARS-CoV-2. Overall, five types of vaccines consisting of messenger RNA (mRNA) vaccine, nonreplicating and replicating viral vectors vaccine, inactivated virus vaccine, protein vaccine, and DNA vaccine have been authorized for clinical use based on different genotypes **(Figure 3)**. In addition, in order to effectively control the spread of the COVID-19 epidemic, other types of vaccines have been under development, such as live attenuated vaccines that have been undergoing animal experiments 6, 37-40. Clinical experimental studies show that each vaccine has a unique immune mechanism, advantages and disadvantages, vaccine efficacy and vaccine effectiveness** (Table 1)**. For example, the mRNA vaccine delivers fragments of mRNA (isolated or genetically engineered) to the cytoplasm through lipid nanoparticles to produce immunoglobulin. The mRNA vaccine has the following advantages: (1) it is easy to manufacture; (2) elicits both antibody and cytotoxic T-lymphocyte responses; and (3) translation of mRNA occurs in the cytosol and not in the nucleus of the host cell. The mRNA vaccine also faces the following disadvantages: (1) booster doses are likely needed; (2) includes modified nucleosides to prevent degradation because mRNA is unstable; (3) a carrier molecule is necessary to enable entry of the mRNA into cells; and (4) cold-chain is required. Therefore, the selection of SARS-COV-2 vaccines should be based on the local epidemiological profile of SARS-COV-2.

As shown in **Table 1**, mRNA vaccines generally have high vaccine efficacy and vaccine effectiveness than other types of vaccines. On the other hand, the viral vector vaccine, Sputnik V, has the highest vaccine effectiveness. In terms of clinical application, the most common method of transport for both mRNA and viral vector vaccines is to use lipid nanoparticle or vectors to deliver the spike-encoding mRNA of SARS-CoV-2 into the human body. Pfizer-BioNTech (BNT162b2) and Moderna (mRNA-1273) vaccines, the most commonly used and first developed mRNA vaccines, were rolled out worldwide in December 2020, which played an important role in controlling the spread of SARS-COV-2 51. Furthermore, the mRNA vaccines were shown to be effective against the first major mutation (D614G) of the original strain and have been proven to effectively reduce hospitalization rate and mortality. Thus, they played a vital role in the developing immune response against the Delta variant by significantly decreasing the reduction of neutralization level 52-57. As viral vector vaccines, Ad26.COV2.S and AZD1222 had extremely high preventive effects on hospitalization caused by Delta variant, and demonstrated stronger neutralizing antibody (NAbTs) against all strains compared to mRNA-based vaccines 58, 59. BBV152 is an inactivated vaccine that can induce antibodies that widely act on the whole epitope of the virus, demonstrating its neutralizing potential 60. The protein vaccine is a virus-like particle or a characteristic protein of SARS-CoV-2 that is synthesized *in vitro*, which can effectively prevent the viral RNA from entering the human body** (Table 1 and Figure 3)**. In summary, each vaccine has its unique advantages, disadvantages, vaccine efficacy and vaccine effectiveness. In actual use, the corresponding selection should be made according to the regional situation.

## The immunity mechanism of COVID-19 vaccines

**Figure 4:**
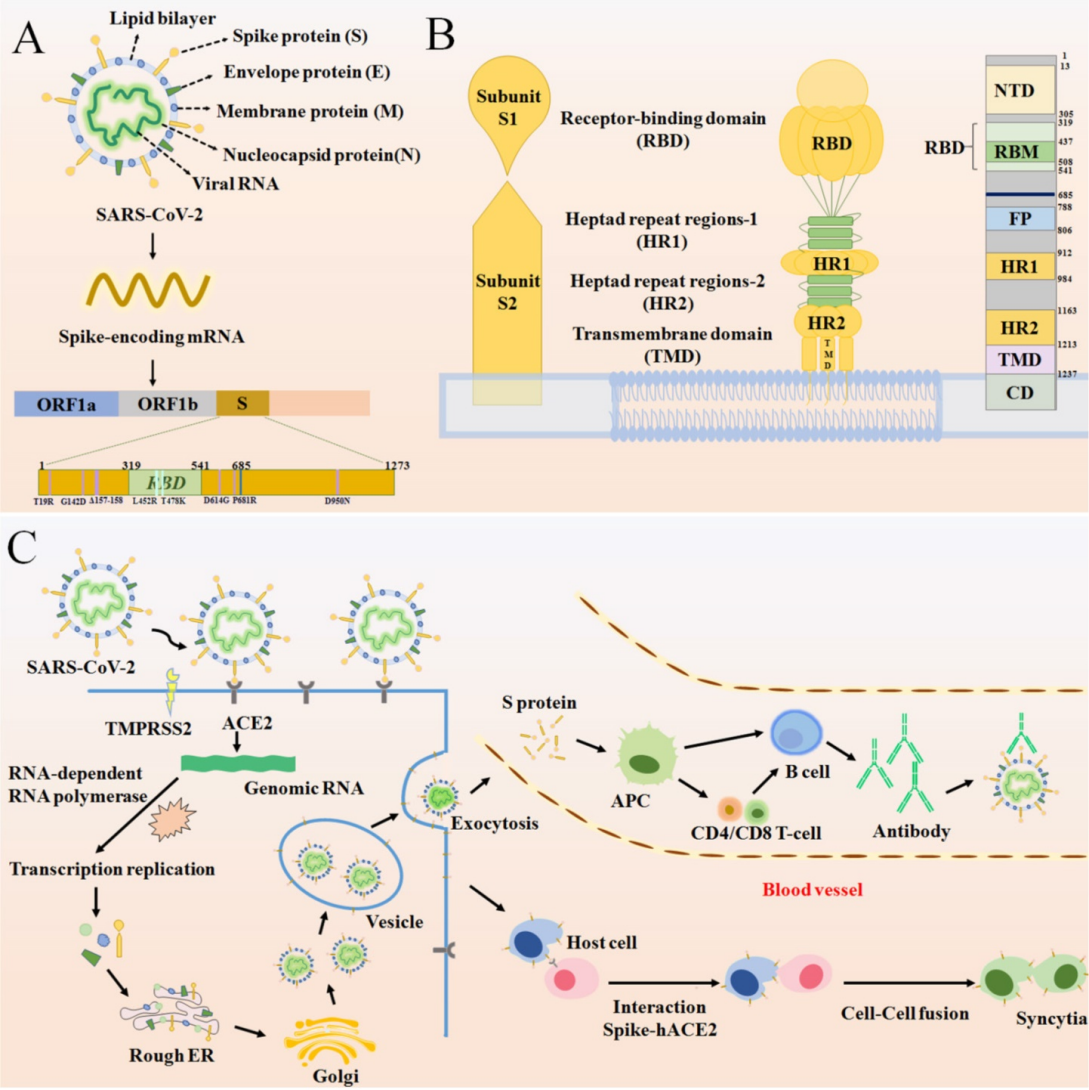
(A) The basic structure of SARS-CoV-2 and the position of some significant mutation in the S protein. (B) A schematic diagram of the SARS-CoV-2 S protein anchoring to the virus envelope to interpret the structural characteristics of S protein from three perspectives. The first diagram depicts the basic components of S protein. The second schematic suggests a model for S protein trimer and the binding principle for membrane fusion. The third is a linear ordering of structural domains located at different positions on the S protein. (C) A schematic of SARS-CoV-2 binding to ACE2 under the catalysis of TMPRSS2 and entering the host cell, forming syncytia and producing an immune response.

Since the identification of SARS-CoV-2 as etiological agent of COVID-19, several attempts have been made in order to prevent infection and disease 61. Overall, vaccines are considered the mainstay in halting and ending the COVID-19 pandemic. Therefore, vaccine development began at a strongly accelerated pace shortly after the beginning of the SARS-CoV-2 outbreak. Yet, there is still only little understanding of the relationships between SARS-CoV-2 infection, antibody responses and protection. Understanding the basic structure of the S protein of SARS-CoV-2 starts from tracing the immune mechanism of the vaccine. The S protein is a transmembrane glycoprotein on the viral envelope in the shape of a homotrimer. It consists of two subunits: spike 1 (S1)and spike 2 (S2), which play an important role in entering host cells 62** (Figure 4A)**. The S1 subunit, consisting of the N-terminal domain (NTD) and receptor binding domain (RBD), is responsible for viral attachment to the host cell through angiotensin-converting enzyme 2 (ACE2) whereas the S2 subunit completes membrane fusion 62-64 **(Figure 4B)**. The RBD domain is an important target for antibodies and many other antiviral drugs because it is a key domain in the S1 subunit, which is used to invade the host cell by binding to ACE2 65-70. Specifically, there is an antiparallel β-sheet at the binding sites between virus and ACE2, which can change its conformation with mutations in or around the RBD interfacial region, thereby increasing the binding affinity. Furthermore, a protease on the surface of the host cell, transmembrane protease serine 2 (TMPRSS2), mediates cleavage of the S2 protein, which causes the viral envelope to fuse with the host cell membrane 71, 72. **(Figure 4C)** 16, 73, 74. It is certified that effective vaccines produce specific antibodies that competitively inhibit ACE2 to neutralize and block the attachment and fusion of the virus to host cells 6, 75. Currently, authorized vaccines can trigger robust humoral and cellular immunity and induce a large number of antibodies to weaken the viral infection 76-79. According to clinical investigations, existing vaccines can effectively target and eliminate factors that cause severe COVID-19 80. However, following the initial surges of the Alpha and the Beta variants, a more infectious Delta variant is now surging, further heightening the health crises caused by the SARS-CoV-2 pandemic. The Delta virus variant, which has led to a sharp rise in cases of breakthrough infections following vaccination, makes it particularly disturbing and concerning. Therefore, currently used vaccines offer protection against known variants of concern, including the Delta variant 81. However, the Delta variant has exhibited some ability to evade the immune system as neutralizing antibodies from prior infections or vaccines are less receptive to binding with the Delta S protein, eventually leading to breakthrough infections.

## The vaccine breakthrough infection status

**Figure 5:**
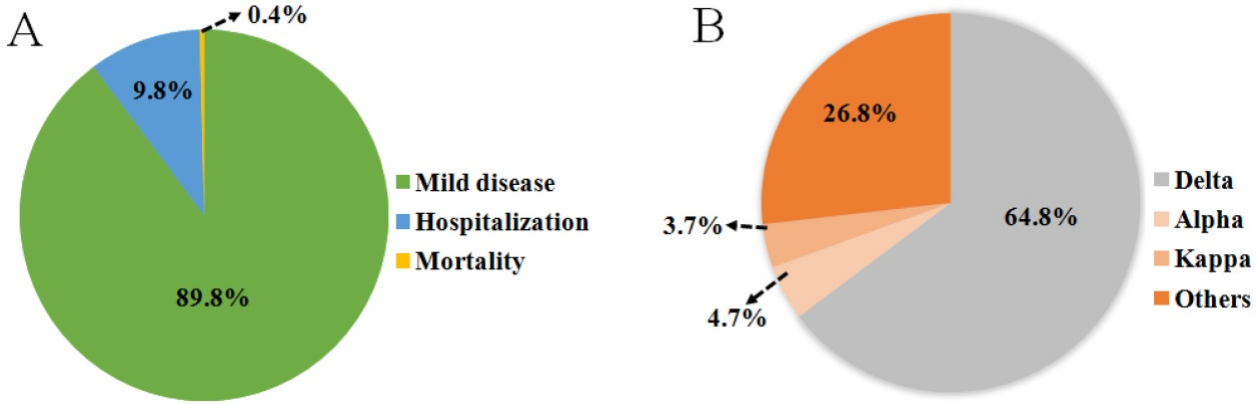
(A) An investigation of the severity of breakthrough infections from India showed that most breakthrough infections were mild diseases and only 10.2% of patients were hospitalized or died. (B) The pie chart presents data from a study on the types of variants infected by positive patients in India from April to June 2021.

Emerging variants, like the Delta variant, not only results in increased transmissibility, morbidity and mortality, but also has the ability to evade detection by diagnostic tests; exhibits decreased susceptibility to treatment including antivirals, monoclonal antibodies and convalescent plasma; and possesses the ability to cause reinfection in previously recovered and vaccinated individuals. SARS-CoV-2 genome is also prone to various mutations that leads to antigenic drift resulting in escape from immune recognition. At present, Delta has been detected to contain mutations T19R, G142D, Δ156-157, R158G, L452R, T478K, D614G, P681R, E484Q/K, and D950N on the RBD of the spike protein 16, 82, 83 **(Figure 4A)**. Among them, L452R may promote the interaction between the RBD and ACE2 receptor, allowing more RNA of virus to enter the host cell by effectively inter-connecting the membranes around the cell and virus **(Table 2)** 71, 84. It has also been proven that P681R is located near the host protease binding site and it is likely the main mutation that drives the cleavage of the S protein and enhances the infectivity of the virus** (Table 2)** 85, 86. Breakthrough infections were first reported in individuals immunized with the Pfizer-BioNTech vaccine in Israel in January 2021. Subsequently, similar cases occurred in individuals vaccinated with the Pfizer and Moderna (mRNA-1273) vaccines in India. Immediately afterwards, breakthrough infections of vaccinated groups occurred in Italy, the United Kingdom, the United States and other countries **(Figure 1)**. During June 2021, Douglas et al. reported an incidence of breakthrough infections of 2.57% in a cohort of 12248 health care workers, among whom 58.5% received at least one dose of the ChAdOx1 nCoV-19 vaccine (AZD1222) in accordance with guidelines from the government of India 87-93. Another scientific team from Israeli observed health care workers who received two doses of the BNT162b2 vaccine. Among them, 0.4% were infected post-vaccination with only mild or asymptomatic cases 94. The surge in reinfection rate further shows that the virus broke through the protective barrier provided by existing vaccines and the threat to public health has also significantly escalated. In addition, clinical data from Indiana University School of Medicine in the USA show that the proportion of breakthrough infections in people who received only one or no doses of vaccination was nearly four times as likely as fully vaccinated individuals and 64% of those breakthrough infections were asymptomatic 87, 93. In India, where breakthrough infections occurred extensively, only 9.8% of breakthrough cases required hospitalization and the mortality rate was only 0.4% **(Figure 5A)**. The probability of breakthrough infections caused by Delta, Alpha and Kappa variants was 64.8%, 4.7% and 3.7%, respectively 15 **(Figure 5B)**. A set of research data on Delta-induced hospitalization and admission to Intensive Care Units (ICU) from the National Centre for Disease Control in Delhi, India showed that 76.83% of cases were above the age of 50 years old where they did not complete the two-dose vaccination regimen and consisted of five or more relevant comorbidities such as hypertension or diabetes mellitus 24. Research from Lu et al. and Hasan et al. indicated that compared with other VOCs, this infection was mainly caused by the high contagiousness of the Delta variant, which had a viral load of approximately 1000 times that of the original strain, followed by the Alpha and Kappa variants 95, 96. Although breakthrough infections are common for the Delta variant, but vaccination can still help prevent severe infections and reduce associated hospitalizations 97, 98. Therefore, the breakthrough infection caused by the Delta Variant poses an unprecedented challenge to the prevention and control of SARS-COV-2.

## The mechanism of vaccine breakthrough

**Figure 6:**
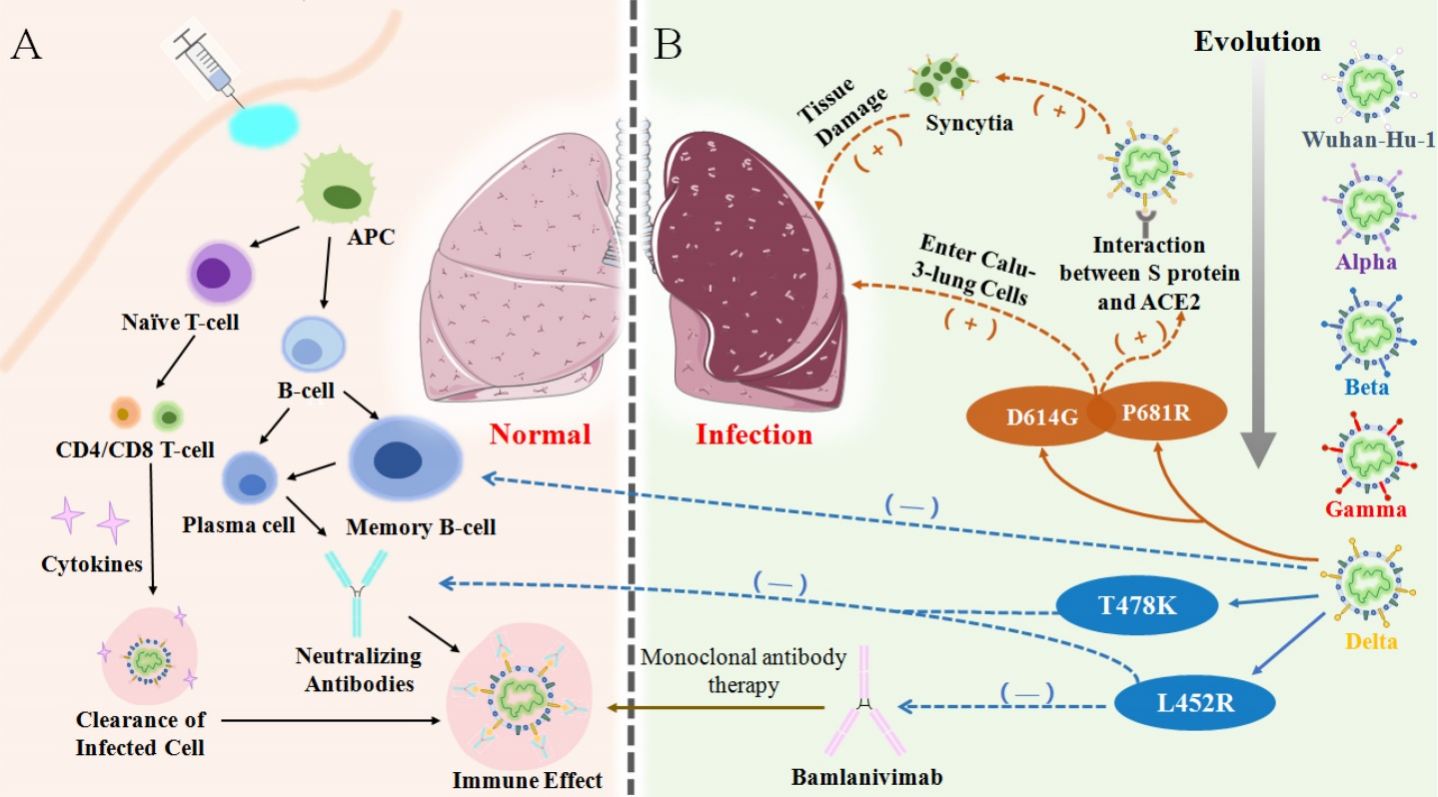
(A) Vaccination or antigen induces the activation of the antigen-presenting cells (APCs) and vaccine-elicited immune responses lead to the clearance of infection in body. (B) Through evolution, the mutation of Delta variant causes immune evasion, the high expression of certain mutations in the lungs causes an increase in virus entering host cells, resulting in severe lung damage.

Upon emergence of Delta variant, the vaccine efficacy of existing vaccines became significantly lower than that of the original strain and previous VOCs, which mainly manifested due to the weakening of neutralization and the failure of monoclonal antibodies 35, 86, 99. An *in vitro* study showed that the Delta variant was less sensitive to neutralizing antibodies in sera from recovered individuals and even showed an 8-fold reduction in sensitivity to vaccine-elicited antibodies 56, 94, 100. The data of Chen et al. suggested that the Delta variant escaped natural-infection-mediated neutralization with an average of 3.2-fold reduction in live virus neutralization assays. Analysis using serum from vaccinated individuals also showed reduction in the neutralization of Beta across different platforms with an average of 7.1-fold reduction for a non-replicating vector platform and 4.1-fold (3.7-4.4) reduction for mRNA platform 86. It can be reasonably inferred that Delta has the ability to avoid immune responses and especially evade humoral immunity 72, 101. At present, mutations on the S protein of the virus are recognized as the main culprit for the invasion of cells and evasion of the immune system **(Figure 6)**. Compared with previous variants, the expanding deletion-prone regions of the NTD and substitution mutations in the RBD led to new denaturation of the S protein 102. There are four major mutations that cause breakthrough infections by Delta. For example, L452R and T478K are two mutations on the RBD which are found to be key mutations in reducing the serum neutralizing antibody level elicited by vaccination or infection 103. P681R is located in the proximity of the S1-S2 cleavage site on spike protein. On the one hand, P681R can induce more tissue damage and increasingly trigger cell-to-cell fusion. On the other hand, the formation of more syncytia contributes to the effective spread of the variant and inter- and intra-host spread, eventually inducing an exponential invasion into host cells **(Table 2)** 103, 104.

Delta was confirmed by Prerna Arora et al. to be able to enter into more Calu-3-lung cells with the assistance of D614G, which may be the main reason for the more prominent pulmonary symptoms of severe infections 3, 116-118. However, severe cases are rarely detected from breakthrough infections because natural infection or vaccination establishes memory immunity and can theoretically provide long-lasting protection driven by memory cells 119, 120. However, there was decreased responsiveness of memory B-cells (MBC) to VOCs in blood samples of vaccinated individuals. Particularly, the frequency of MBC responsiveness to VOC RBDs was significantly lower compared to homologous RBDs 121, 122. Neutralizing antibodies produced by the MBC and associated with T478K and L452R mutations were also reduced 36. The L452R mutation was reported to be related to the resistance of the Delta variant to Bamlanivimab, one of the monoclonal antibodies. In accordance, failure of the memory immune system as well as failure of the entire humoral immune system has led to the ferociously spread of breakthrough infections in the world **(Figure 6)**.

## What is the vaccination strategy in future?

At present, widespread vaccination to achieve herd immunity is the only effective way to prevent COVID-19, otherwise SARS-COV-2 would circulate long-term 54. Though the WHO has tried to balance the arrangement of vaccines around the world, there is still an inequitable distribution in low-income countries where there are minority groups that have only received at least one dose. However, the rate of evolution of virus can only be controlled only if a rational vaccination strategy is formulated 123, 124. It is thus pertinent to examine research results on vaccines, which may bring to light methods for better vaccination strategies.

Firstly, there is a higher likelihood of novel variants to emerge in high-risk populations. According to the suggestions from WHO, concessions should be made to low-income groups for first doses of SARS-CoV-2 vaccines, though it is the imperative to eventually complete the two or even three dose regimens 93, 125. For people with co-morbidities and of disease-prone ages, there are several vaccines that are confirmed to be safe and effective for adolescents, the elderly and chronic patients, such as BNT162b2 (Pfizer; BioNTech) and Sinovac's mRNA vaccine CoronaVac 126. Furthermore, vaccine breakthrough clusters among vaccinated healthcare workers (HCW) are of concern, where a transmission chain dominated by Delta variant can occur 2. Thus, further research and development of targeted vaccines for wider groups are needed.

Secondly, the scientific community speculates widely that existing vaccines may have little validity on nasal infections, since the SARS-CoV-2 virus in most breakthrough infection patients were detected in the upper respiratory tract with mild or no symptoms 90. As follows, basic research in superior mucosal vaccines should be developed in order to generate tissue resident memory B cells in the respiratory mucosa and to enhance the efficacy for mediating humoral immunity in the respiratory tract 12, 90. Presently, a T cell-based adoptive cellular immunotherapy is expected to hinder the combination between AXL and NTD, which is associated with viral entrance into the host cells of vaccinated individuals 84.

Lastly, regarding treatment of infected patients, there are numerous research directions that are worth exploring in depth. Monoclonal antibodies (mAbs) have been used to be as a valid therapy for COVID-19 patients. Studies showed that CTP59, a monoclonal antibody, had improved efficacy against Delta, Epsilon, and Kappa variants, could evade drug resistance and become a curable option to viral infections with identical mutation sites, though varying mutations have been revealed to compromise its therapeutic effect 22, 71, 127-129. In addition, there are currently more than 1,000 studies on antiviral drugs as potential treatments, including small molecular drugs such as 3CL protein inhibitors and RNA synthesis inhibitors 130. Miao Cao et al. successfully designed a series of peptide-based pan-CoV fusion inhibitors, which are highly effective in inhibiting infection against pseudotyped human coronaviruses 131. Moreover, the increase in transmissibility was reported to be more so caused by viral load or probability of infection rather than environmental stability 104. In this regard, the authors theorized that stricter hygienic and behavioral measures would comparatively not have a significant impact on probability of infection as compared to wearing masks indoors. Therefore, it is essential to cultivate awareness and form appropriate strategies in preventive measures.

## Conclusion

The Delta variant of SARS-CoV-2 has become one of the most worrisome variants thus far during the pandemic and has been rapidly spreading worldwide, making it responsible for the recent surges in infections and deaths. Although current vaccines are still shown to be protective against this variant, it is also becoming clearer that the variant can evade the immune system by rendering neutralizing antibodies from prior infections or vaccination less sensitive to binding with the spike protein. In short, existing vaccinations do not block Delta variant, but the efficacy of vaccines to slow down the evolution of virus is credible as outcomes show an obvious advantage in averting severe symptoms, hospitalization and deaths. In addition, vaccine breakthrough cases are often undercounted and fully vaccinated populations should still practice preventive measures. Along with the possible emergence of various SARS-CoV-2 variants in the future, there is a predictable challenge to develop targeted vaccines against mutations on the S protein and terminate the COVID-19 pandemic as soon as possible. Therefore, aside from a series of vaccine roll-out measures, continuous monitoring of post-vaccination breakthrough infections must be adopted by all countries.

## Figures and Tables

**Table 1:** The main mechanism, characteristics, efficacy and effectiveness of the major types of vaccines.

Type	Vaccine (Country)	Mechanism	Advantages and Disadvantages	Vaccine efficacy (95% CI)	Vaccine effectiveness (95% CI)	Ref
mRNA Vaccines	mRNA-BNT162b2 (China)	The mRNA fragments that can produce viral antigens in the cytoplasm by directly protein translation from the spike-encoding mRNA *in vivo*.	**Advantages:** •Easy to manufacture. • Induces antibody and cytotoxic T-lymphocyte responses. **Disadvantages:** • Booster dose may be required. • Contains modified nucleosides to prevent degradation because the mRNA is unstable. • Carrier molecules are necessary to allow messenger RNA to enter cells. • Cold-chain required.	95% (90.3-97.6)	93.0% (95%CI 92.6-93.4)	41
	mRNA-1273 (United States)			94.1% (89.3-96.8)	90% (68-97)	42
Viral vector	ChAdOx1_nCoV-19 (England)	Replicative or non-replicative viral vectors that transport viral genes without contacting with human immune system.	**Advantages:** • Single dose possible. • Easy to manufacture. • Induces antibody and cytotoxic T-lymphocyte responses. **Disadvantages:** • The vector is considered a genetically modified organism with potential risks to the environment. • Potential safety issues for immunocompromised patients. • Host immunity to the viral vector (a person who has antibodies) may reduce the effectiveness of the vaccine.	74.0 (65.3 to 80.5)	79% (95% CI 65 to 88%)	43, 44
	Sputnik Light (Russia)			unpublished	78.6-83.7%	44
	Ad26.COV2.S (United States)			66.9% (59.1-73.4) at ≥ 14 days and 66.5 (55.5-75.1) at ≥ 28 days	76.7% (30.3-95.3)	45, 46
	Ad5-nCoV (China)			65-69%	unpublished	44
	Sputnik V (Russia)			91.6% (85.6-95.2)	97.8%	47
Inactivated vaccine	BBIBP-CorV (China)	The virus is physically or chemically inactivated, but the virion remains intact. It can induce a specific immune response to the S protein without causing infection.	**Advantages:** • Impaired immune memory. • Safer than live attenuated vaccines. • Naturally stimulates the immune system without adjuvants. **Disadvantages:** • Impaired immune memory. • Short duration of immune response (booster may be required). • Safety testing is often needed to ensure that live attenuated viruses do not easily revert to the wild-type. • Low production efficiency.	78.1% (64.8%-86.3%)	unpublished	48
	COVIV (China)			72.8% (95%CI:58.1-82.4)	unpublished	48
	CoronaVac (China)			83.5% (65.4-92.1)	67% (65-69)	49
	Covaxin (India)			78% (95%CI: 61-88%)	unpublished	50
	QazVa (Kazakhstan); KoviVac(Russian); SARS-CoV-2 Vaccine (China); COVIran Barakat (Iran)			unpublished	unpublished	49
Protein subunit	Soberana 02 (Cuba)	Recombinant viral proteins induce an immune response that induces neutralizing antibodies in the absence of a cell-mediated response.	**Advantages:** • Antibody response is expressed. • Techniques already used for many viral diseases. • Lower production costs because only part of the pathogen needs to be produced. **Disadvantages:** • Booster dose may be required. • Adjuvants are needed to stimulate the immune system.	62%	unpublished	49
	Abdala (Cuba)			92.28%	unpublished	49
	Zifivax (China); EpiVacCorona (Russian)			unpublished	unpublished	49
DNA Vaccines	ZyCoV-D (India)	Viral antigens encoded by recombinant DNA plasmids are produced in host cells through a continuous transcription-translation process.	**Advantages:** • It can be produced simply and quickly by PCR or synthesis • Easy mass production • Safer than others,methods, such as inactivated virus vaccines • More heat resistant **Disadvantages:** • Expensive. • Booster dose may be required. • Potential integration with the human genome.	unpublished	unpublished	49

**Table 2:** Four major spike protein mutations, structural effects, viral and antibody neutralization effects were identified in the B.1.617 lineage

Mutation	Location on the mRNA	Effect on structural aspects	Effects on transmission and/or infectivity of SARS-COV-2	Antibody neutralization titer	Ref
L452R	RBD	Decreased binding to select mAb	Enhanced spike stability, viral infectivity, viral fusogenicity, and thereby promotes viral replication.	Neutralization titers were 4.0-6.7 times lower in recovered patients and 2.0 times lower in vaccinated patients.	105-107
T478K	RBD	Enhanced ACE2 binding	May affect the affinity with human cells and therefore influence viral infectivity	T478K substitution reduced the neutralization activity	108
D614G	S1 protein	Enhanced ACE2 binding	Enhanced infectivity, fitness, and transmissibility	The antibody neutralization titer decreased by 2.05 times	109-115
P681R	Near the cleavage site between S1 and S2	Enhanced binding to furin	enhanced viral replication fitness and and accelerates viral fusion	unpublished	36

**Note:** mAb: Monoclonal antibody; ACE: Angiotensin converting enzyme
